# Pattern Recognition Receptor-Mediated Chronic Inflammation in the Development and Progression of Obesity-Related Metabolic Diseases

**DOI:** 10.1155/2019/5271295

**Published:** 2019-09-08

**Authors:** Lili Yu, Yanhua Li, Cancan Du, Weidong Zhao, Hanxiao Zhang, Yun Yang, Aiping Sun, Xiangfeng Song, Zhiwei Feng

**Affiliations:** ^1^School of Basic Medical Sciences, Xinxiang Medical University, Xinxiang 453003, China; ^2^Institute of Precision Medicine, Xinxiang Medical University, Xinxiang 453003, China; ^3^School of Laboratory Medicine, Xinxiang Medical University, Xinxiang 453003, China

## Abstract

Obesity-induced chronic inflammation is known to promote the development of many metabolic diseases, especially insulin resistance, type 2 diabetes mellitus, nonalcoholic fatty liver disease, and atherosclerosis. Pattern recognition receptor-mediated inflammation is an important determinant for the initiation and progression of these metabolic diseases. Here, we review the major features of the current understanding with respect to obesity-related chronic inflammation in metabolic tissues, focus on Toll-like receptors and nucleotide-binding oligomerization domain-like receptors with an emphasis on how these receptors determine metabolic disease progression, and provide a summary on the development and progress of PRR antagonists for therapeutic intervention.

## 1. Introduction

Dramatic rises in obesity and related disorders have occurred globally in just decades, sparing no age groups. The prevalence of obesity among adults has tremendously increased in the past four decades. Approximately 2 billion adults worldwide are overweight, and among them, more than half a billion are obese (World Health Organization, 2018). The development of obesity is closely associated with serious metabolic disorders, such as insulin resistance, type 2 diabetes mellitus (T2DM), hepatic steatosis, and cardiovascular disease, which cause significant morbidity and mortality worldwide [1]. These kinds of chronic diseases are an enormous burden for individuals, families, and societies, as the quality of life is ruined and treatment requires considerable financial supplies and health care resources. The mechanism underlying the pathogenesis of these diseases should be determined, and an effective approach for relieving and healing should be developed.

Accumulating evidence indicates that obesity is associated with chronic low-grade inflammation, which is the key point in the initiation and progression of obesity-related metabolic diseases, especially insulin resistance, T2DM, nonalcoholic fatty liver disease (NAFLD), and atherosclerosis [2–5]. Inflammation occurs due to obesity, and considerable studies have shown that it may play a decisive role in homeostasis [6, 7]. Thus, the influence of obesity-related inflammation in the initiation and regulation of these diseases is a matter of significance [8]. The immune and metabolic systems are closely integrated and complementary [9–11]. The innate immune system constructs the first line of defense to detect and sense the majority of components elicited by infection and endogenous molecules. Thus, excessive metabolic proteins and metabolites associated with obesity can be recognized by innate pattern recognition receptors (PRRs) [12].

Several PRR subfamilies, such as Toll-like receptors (TLRs), retinoic acid-inducible gene I-like receptors, nucleotide-binding oligomerization domain- (NOD-) like receptors (NLRs), C-type lectin receptors, and DNA sensors have been identified [13]. PRRs recognize pathogen-associated molecular patterns (PAMPs) induced by gut microbiota and infection and danger-associated molecular patterns (DAMPs) caused by metabolic stress or tissue damage to activate innate immune responses and lead to the expression of diverse arrays of downstream signaling pathways [14, 15]. TLRs and NLRs are the two most characterized and described innate receptors in the progression of metabolic diseases, which induce downstream intracellular signaling cascades to produce inflammators such as cytokines, chemokines, and costimulatory molecules. TLRs predominantly recognize the extracellular or endosomal compartments, whereas NLRs sense invading intracellular pathogens and perturbations associated with stress or damage [16, 17].

Sustained activation and uncontrolled regulation of PRR-mediated innate immune responses can lead to chronic inflammation, which promote the development and progression of many chronic diseases. Genetic, biochemical, and clinical studies have indicated the close link between PRRs and the risk of many chronic diseases. This review summarizes and discusses the recent advancements in understanding the role of PRRs and their downstream signals in the pathogenesis of prevalent obesity-associated diseases.

## 2. Obesity-Induced Chronic Inflammation in Metabolic Tissues

**Figure 1:**
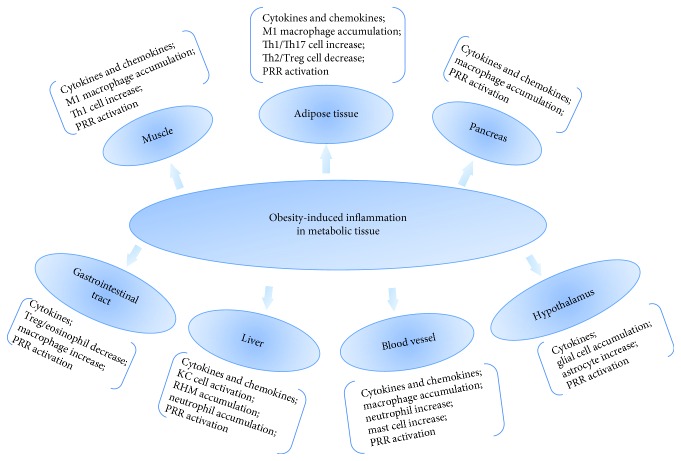
Obesity-induced chronic tissue inflammation states in metabolic tissues. Chronic tissue inflammation induces a range of effects on adipose tissue, muscle, liver, pancreas, gastrointestinal tract, blood vessel, and hypothalamus. These inflammatory changes include the secretion of cytokines and chemokines, infiltration of immune cells, and activation of PRRs, which are the key points in the initiation and progression of obesity-related metabolic diseases.

Multiple PRRs have been implicated in the recognition of metabolic stress and initiation of inflammatory responses in various tissues, which contribute to the development of metabolic diseases [18, 19]. Metabolic syndrome-associated chronic inflammation is related to multiple organs and tissues, including adipose tissues, pancreas, liver, muscle, blood vessel, hypothalamus, and gastrointestinal tract (Figure 1).

### 2.1. Adipose Tissue

Adipose tissue inflammation is considered a crucial event that leads to metabolic disease. The first hint is the increased expression and production of tumor necrosis factor- (TNF-) *α* in adipose tissue of obese individuals and its direct role in obesity-induced insulin resistance [20]. However, TNF-*α* antagonism does not show a significant improvement on insulin sensitivity in patients with metabolic syndrome or T2DM [21]. Accumulated data have confirmed other upregulations of proinflammatory cytokines and chemokines in enlarged adipose tissue sites of obese individuals and those with T2DM, such as interleukin- (IL-) 1*β*, IL-6, and monocyte chemotactic protein- (MCP-) 1. One study suggests that galectin-3-mediated inflammation induces insulin resistance [22].

Adipose tissues normally contain multiple immune cells that together maintain the integrity and metabolism of adipocytes [23]. Under normal conditions, immune cells in adipose tissues operate in an anti-inflammatory state and mainly contain M2-resident macrophages, CD4^+^ T helper (Th) 2, and CD4^+^ regulatory T (Treg) cells, which secrete anti-inflammatory IL-10 and Th2 cytokines. In obesity, adipose tissues are associated with a marked accumulation of proinflammatory immune cells, with M1-polarized macrophages having the most abundant population. Meanwhile, the number of CD4^+^ Th1 cells and Th17 cells is increased, which secrete interferon- (IFN-) *γ* and IL-17 as major proinflammatory cytokines in obese adipose tissues, whereas the number of Treg and Th2 cells is decreased [24]. In addition, the infiltration of CD8^+^ T cells and neutrophils is assumed to promote the recruitment and activation of macrophages, and B cells are implicated in producing pathogenic antibodies and proinflammatory cytokines contributing to chronic low-grade inflammation in obesity [25].

However, the molecular events and precise triggers of obesity-associated inflammation in adipose tissue are still not fully clear. One potential mechanism is that underlying elevated lipid metabolites can trigger the activation of PRRs, including saturated long-chain fatty acids (SFAs), ceramides, and modified low-density lipoproteins (LDLs) [1]. PRRs can also be triggered by the production of other endogenous damage-associated signals during the development of metabolic diseases, such as high mobility group box 1 (HMGB1) and fetuin A [16].

### 2.2. Pancreas

Inflammation in pancreatic islets contributes to *β*-cell dysfunction and apoptosis, which results in reduced insulin secretion [26]. As in adipose tissue, macrophages infiltrate and accumulate in pancreatic islets of obese T2DM individuals and diet-induced obesity mice, which produce proinflammatory cytokines and chemokines, such as TNF-*α*, IL-1*β*, and MCP1 [27]. The most well-studied mechanism by which islet macrophages cause *β*-cell dysfunction is through the secretion of IL-1*β*, and interference with the IL-1 pathway has been demonstrated to relieve T2DM and restore *β*-cell function in humans [28, 29]. A recent study indicated that two islet-resident macrophage populations, namely, intra- and peri-islet macrophages, exist in mice pancreas, and obesity induces the local expansion of resident intraislet macrophages, independent of recruitment from circulating monocytes [30]. Functionally, intraislet macrophages impair *β*-cell function in a cell-cell contact-dependent manner [30]. PPRs have also been reported in the process of pancreas inflammation. Islet amyloid polypeptide (IAPP), a protein that forms amyloid deposits in the pancreas during T2DM, can activate nucleotide-binding and oligomerization domain (NACHT), leucine-rich repeat (LRR), and prin domain- (PYD-) containing protein 3 (NLRP3) in causing infiltrated macrophages, generating IL-1*β*, and reducing *β*-cell mass [31].

### 2.3. Liver

Hepatic steatosis is a common feature in obesity that can result in NAFLD and ultimately progress to nonalcoholic steatohepatitis (NASH) and fibrosis. The inflammatory cytokines derived from immune cells in the liver can potentiate steatosis. Two major kinds of macrophages, namely, Kupffer cells (KCs) and recruited hepatic macrophages (RHMs), are found in the liver. KCs are resident macrophages and are important for hepatic inflammatory states [32]. During obesity development, steatotic hepatocytes and KCs release chemokines such as MCP1 to recruit circulating monocytes. RHMs are abundantly accumulated in the liver, and they are highly proinflammatory and characteristically express various cytokines, such as TNF-*α*, IL-1*β*, and IL-6. One study indicated that chemotactic eicosanoid LTB4 promotes insulin resistance in obese mice by acting on macrophages, hepatocytes, and myocytes [33]. Similarly, neutrophils accumulate in the liver during obesity and release a similar set of cytokines and chemokines contributing to the overall inflammatory process. A combination of hepatic insults is proposed to drive hepatic steatosis pathogenesis and activate various innate immune pathways. TLRs and NLRs are widely expressed in multiple hepatic cells and KCs. Intrahepatic activation of PRR signaling by endogenous and pathogen-derived ligands plays a critical role in the development of hepatic steatosis and NAFLD [34].

### 2.4. Muscle

Skeletal muscle (SM) insulin resistance plays a key role in metabolic dysfunction. One possible etiologic factor that causes decreased muscle insulin sensitivity is inflammation. Numerous studies have demonstrated the increase of inflammatory markers in the SM of obese and diabetic individuals, which includes the activation of proinflammatory cytokines in muscle cells and circulating cytokines derived from other tissues. These muscle-derived mediators may also contribute to low-grade systemic inflammation with obesity. The first cytokine produced by the muscle fiber is IL-6, which is reported to accumulate in the bloodstream in response to muscle contraction and is shown to improve insulin sensitivity. However, long-term and chronic exposures to IL-6 during obesity can cause insulin resistance and inflammation in muscle tissues [35].

Similar to adipose tissue, resident macrophages are located in the epimysium and perimysium of SM. In obesity, many proinflammatory immune cells, including M1 macrophages and Th1 cells, infiltrate and accumulate in muscle tissues, which secrete proinflammatory cytokines, such as TNF-*α*, IL-1*β*, and MCP1 [36]. This infiltration may be consecutive to chemokine release by resident macrophages and/or myocytes themselves in response to PRR-mediated recognition of PAMPs or DAMPs. Similar to adipose tissues, SM tissue is sensitive to lipopolysaccharide (LPS) and circulating SFAs, inducing the production of cytokines TNF-*α*, IL-6, and chemokines depending on PRRs [37].

### 2.5. Blood Vessel

Atherosclerosis is a chronic inflammatory disease of the blood vessels; it is a common cardiovascular disease associated with lipid accumulation and plaque formation in the blood vessel wall [23]. Atherosclerosis is promoted through obesity-associated hyperlipidemia and inflammation. At the initiation of atherosclerosis, circulating LDL particles are detained by subendothelial cells and oxidized by oxygen radicals and enzymatic attacks. Then, the modified LDLs activate innate immune receptors and trigger inflammatory cascades to induce the expression of inflammatory cytokines, chemokines, and costimulatory molecules. Inflammation cytokines such as TNF-*α*, IL-1*β*, and IL-18 are elevated in atherosclerosis and associated with disease severity. Monocytes, dendritic cells, and T cells are recruited into the intima by these chemokines. Many monocytes are differentiated into macrophages and eventually are transformed into foam cells as cholesterol accumulation. Neutrophils and mast cells are also recruited to atherosclerotic lesions, which produce proatherogenic mediators that contribute to lesion growth and disease progression [38]. Various PRRs are expressed in arterial lesions and activated by oxidized LDL particles, fibronectin, cholesterol crystals, and calcium crystals during atherosclerosis progression [39].

### 2.6. Hypothalamus

The hypothalamus plays an important role in the homeostatic regulation of body weight through energy intake and expenditures. Increased hypothalamic glial cell and/or astrocyte accumulation occurs during obesity [40]. Studies have confirmed that the expression of inflammatory cytokines is increased through PRRs in obesity, which is associated with hypothalamus insulin resistance. In particular, clinical and experimental studies have identified resistin as a key hormone linking obesity-induced hypothalamic inflammation and insulin resistance through the activation of TLR4 signaling pathways [41].

### 2.7. Gastrointestinal Tract

The development of obesity and related metabolic diseases is associated with altered gastrointestinal microbiota composition [42]. Microbiota can produce metabolites and byproducts to induce proinflammatory cytokines and modulate metabolism in the development of obesity, tissue inflammation, and metabolic dysfunction. Microbiota transplantation from obese humans into lean recipients leads to an increase in systemic insulin resistance [43]. Mice with high-fat-diet- (HFD-) induced obesity display altered immune cell populations in the intestinal lamina propria, including a reduced proportion of Treg cells and eosinophils and elevated macrophages [44]. Obesity could induce increased gastrointestinal permeability and endotoxemia, resulting in high levels of circulating metabolites and byproducts produced by microbiota [45]. Microbes and their products contain PAMPs, which are recognized by the host innate immune system through PRRs, including LPS, bacterial DNA, and peptidoglycan (PGN).

## 3. TLRs and Obesity-Related Metabolic Diseases

TLRs execute an irreplaceable role in controlling the inflammation of different metabolic organs in obesity-induced metabolic diseases. As widely expressed and highly conserved transmembrane receptors, TLRs are at the intersection of diet and metabolism and may therefore be important determinants of obesity and metabolic diseases [18, 46]. TLRs are type I transmembrane domain proteins with a tripartite structure: an amino- (N-) terminal extracellular domain as ligand recognition domains, a signal transmembrane spanning region, and a carboxyl- (C-) terminal cytoplasmic signaling domain [47]. Broadly, TLRs use two main adaptors, namely, myeloid differentiation primary response gene 88 (Myd88) and TIR-domain-containing adaptor-inducing IFN-*β* (TRIF), to mediate the downstream signaling pathways.

**Figure 2:**
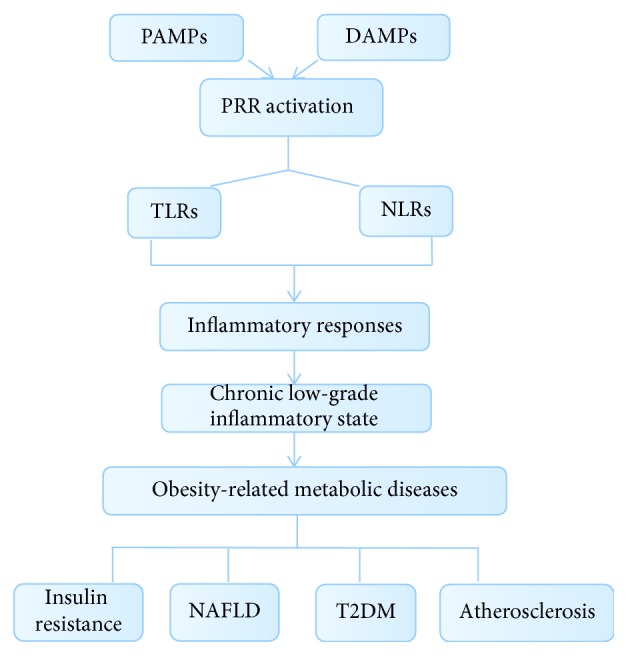
Overview of PRRs in linking inflammation. As important players of the innate immune system, PRRs sense DAMPs from metabolic stress and/or tissue damage and PAMPs from gut microbiota and/or infection. Activation of PRRs induces a series of inflammatory responses and systemically mediates a chronic, low-grade inflammation, which promotes the pathogenesis of obesity-related metabolic diseases, such as insulin resistance, NAFLD, T2DM, and atherosclerosis.

With the exception of TLR3, all TLRs require the engagement of Myd88 to induce the activation of NF-*κ*B, activator protein-1, mitogen-activated protein kinases (MAPKs), and the IFN regulatory factor- (IRF-) 7 pathway, which lead to the expression of cytokines and IFNs. In addition, TLR3 and TLR4 engage TRIF to trigger a specific signaling cascade that results in the activation of NF-*κ*B, MAPKs, and IRF3 to induce the production of proinflammatory cytokines and type I IFNs [48]. To date, at least 13 TLRs have been identified in humans and mice. Multiple TLRs, including TLR2, TLR3, TLR4, TLR5, TLR8, TLR9, and TLR10, have been indicated in the regulation of PAMPs from gut microbiota and DAMPs from metabolic stress and tissue damage, which initiate the inflammatory responses contributing to the development of obesity-related chronic diseases (Figure 2).

### 3.1. TLR2

TLR2 is located at the surface of the cell and forms dimers with other TLRs to recognize the largest range of ligands including free FAs (FFAs), lipopeptides, lipoteichoic acids, lipids, porins, lipoarabinomannan, zymosan, and lipomannan from bacteria and fungi [47]. Elevated plasma FFA levels can account for a large part of insulin resistance in obese patients with T2DM [49]. An in vitro study of human cells showed that TLR2 mediates FFA-induced proinflammatory responses in cultured adipocytes and monocytes/macrophages [50–52].

Human cross-sectional studies found that TLR2 activation and expression are enhanced in adipose tissue and peripheral monocytes of obese subjects compared with lean control [53]. Other studies showed that TLR2 expression is increased in patients with diabetes [54], and TLR2/TLR4 stimulation induces an enhanced inflammation in obese patients with atherosclerosis [55]. Although TLR2 is elevated in obese patients compared with healthy controls, it is unaltered in patients with limited liver disease compared with obesity-related NASH in two clinical studies [56, 57]. In addition, a clinical intervention showed that a one-week HFD leads to reduced TLR2 expression in young healthy men [58]. This discrepancy may be due to the short period of HFD intervention, and further clinical trials are required to confirm this finding.

TLR2 has been implicated in the pathogenesis of obesity and insulin resistance in dietary mouse models. The expression of TLR2 and TNF-*α* was increased in visceral adipose tissue by a HFD as a development of insulin resistance [59]. In a HFD-induced mouse model, macrophages infiltrate hypertrophic adipose tissue and are activated by FFAs via the TLR2 and JNK pathways [60]. In loss-of-function animal studies, TLR2-deficient mice fed with a HFD are associated with the suppression of adipocyte hypertrophy, decreased macrophage infiltration and inflammatory cytokine expression in adipose tissue, and reduced expression of inflammatory cytokines in the liver and pancreas [61–63]. In addition, TLR2-deficient mice are protected from HFD-induced adiposity, insulin resistance, impaired insulin secretion, and hypercholesterolemia [62, 64]. TLR2-deficient mice are also protected from choline-deficient L-amino-acid-defined diet-induced liver inflammation and diet-induced NASH [65]. Furthermore, TLR2 deficiency in apolipoprotein E- (ApoE-) deficient and LDL receptor- (LDLR-) deficient mice reduces atherosclerotic plaque formation in the progression of atherosclerosis [66, 67].

However, another study indicated that TLR2-deficient mice are protected from HFD-induced insulin resistance under germ-free conditions, whereas TLR2-knockout mice show a phenotype of metabolic syndrome under conventional conditions due to the specific role of gut microbiota and increased LPS absorption [68]. TLR2 deficiency in mice is conventionally associated with an increased abundance of *Firmicutes* and a decreased abundance of *Bacterodates* compared with controls [68]. By contrast, another group found that the impact of TLR deficiency on the composition of gut microbiota is minimal under homeostatic conditions [69]. In addition, TLR2-deficient mice are not protected from methionine- and choline-deficient diet- (MCDD-) induced steatohepatitis [70]. This condition is also related to gut microbiota. MCDD increases the amount LPS-producing bacteria, and increased LPS plasma levels could be due to the activation of TLR4 in TLR2-deficient mice. Further studies are needed to elucidate the effect between the TLR2 signaling pathway and gut microbiota.

In a therapeutic animal study, TLR2 suppression using an antisense oligonucleotide improves insulin sensitivity and signaling in muscle and white adipose tissue of mice fed with a HFD [71]. Blockade of TLR2 in intact aortas using an anti-TLR2 antibody attenuates an increase in vascular contraction in rats with streptozotocin-induced diabetes [72]. Another small molecule, C_16_H_15_NO_4_ (C29), was identified as a potential TLR2 inhibitor, which reduced TLR2-induced inflammation [73]. Taken together, although there are some uncertain results, TLR2 inhibition is beneficial to suppress sustained inflammation.

### 3.2. TLR3

TLR3 plays a crucial role in survival by recognizing various viral components, such as double-stranded RNA (dsRNA) and poly(I:C), which is a synthetic dsRNA polymer [74]. Upon recognition, TLR3 uses TRIF as an adaptor to induce the activation of IRF3 and increase the production of type I IFNs for increasing cellular antiviral defenses.

Studies of adipocytes show that TLR3 is present in human adipose-derived stem cells [75] and mature adipocytes [76]. Similarly, the expression of TLR3 is significantly higher than that of the nonadipocytes in the adipose tissue of mice [77]. In a human cross-sectional study, the expression of TLR3 was decreased in obese patients compared with nonobese subjects, and decreased TLR3 was observed in hyperplastic adipose tissue, blood, and inflamed adipocytes [78]. Although TLR3 is highly expressed in adipocytes, the contribution of TLR3 to insulin resistance is enigmatic. In an in vitro study of human cells, defects in TLR3 expression and latent endoribonuclease activation lead to decreased mitochondrial manganese superoxide dismutase expression and insulin resistance in muscle cells of obese individuals [79]. However, in a correlational study of humans, TLR3 expression in adipose tissue from 80 healthy donors did not correlate with BMI or insulin sensitivity (HOMA-IR) [76].

Few studies have indicated the importance of TLR3 in regulating glucose metabolic responses and insulin levels in rodents. In loss-of-function animal models, although TLR3-deficient mice fed with a HFD developed obesity, they exhibited improved glucose tolerance and reduced liver steatosis compared with wild-type (WT) obese mice [80]. Conversely, another study indicated that TLR3-deficient mice with HFD-induced obesity exhibit increased glucose tolerance and serum insulin and decreased serum levels of very low-density lipoproteins and triglycerides (TG) [81]. However, another study showed that TLR3 deficiency in mice does not significantly influence HFD-induced obesity and system insulin sensitivity or inflammation [76]. The impact of TLR3 on metabolic regulation remains unclear. TLR3 appears to play a redundant role in obesity-induced inflammation and insulin resistance, which should be investigated.

### 3.3. TLR4

TLR4 is the most widely described PRR in obesity-induced chronic inflammation and development of metabolic diseases. TLR4 is broadly expressed in human adipocytes, hepatocytes, and monocytes [82–84]. TLR4 responds to ligands such as LPS and initiates its response by forming a complex with MD-2, leading to the activation of the Myd88 and TRIF pathways. Similar to TLR2, TLR4 signaling leads to the production of proinflammatory cytokines, including TNF-*α*, IL-1, and IL-6. Studies of human cells showed that SFAs, including lauric acid, palmitic acid, and stearic acid, activate TLR4 in adipocytes and macrophages in vitro [85]. HMGB1 has been shown to mediate TLR4 activation in human adipocytes, which are also elevated in obesity [82, 86].

Human cross-sectional studies showed that TLR4 expression is increased in adipose tissue and monocytes of obese or diabetic patients, which is correlated with the severity of insulin resistance [53, 87, 88]. Individuals with T2DM show increased cellular membrane levels of TLR4 in monocytes and higher serum concentrations of IL-1*β*, IL-6, IL-8, and TNF-*α* compared with controls [53]. In another cross-sectional study, TLR4 mRNA levels were significantly increased in subjects with NASH compared with obese and NAFLD patients; this occurs within the setting of increased LPS and FAs [83]. A previous study also indicated that TLR4 is a new target for chemoprevention of hepatocellular carcinoma in obesity and steatohepatitis [89]. Human cross-sectional studies showed that TLR4 is involved in monocyte activation of patients with accelerated forms of atherosclerosis, and the upregulation of TLR4 on human monocyte subsets is closely associated with coronary plaque vulnerability in patients [90, 91].

In loss-of-function mouse models, TLR4-deficient mice are protected from diet-induced obesity due to the decrease in proinflammatory cytokine levels [92, 93]. TLR4-deficient mice fed with a HFD exhibit improved insulin resistance, reduced tissue inflammation, and decreased M1 macrophage infiltration [93–95]. Hepatocyte-specific TLR4 deficient mice are also protected from obesity and insulin resistance [96]. In this mouse model, inflammatory cell infiltration was suppressed in the liver and adipose tissue, which indicates that hepatocyte TLR4 signaling produces chemokines to recruit inflammatory cell infiltration. However, macrophage-specific TLR4-deficient mice are not protected from obesity or insulin resistance induced by a HFD [96]. These results suggest that TLR4 expression in hepatocytes plays a decisive role in the initiation of inflammatory responses. In other mouse models, TLR4 deficiency in atherosclerosis-prone ApoE-deficient mice fed a HFD showed significant reduction of aortic plaque areas, plaque lipid content, macrophage infiltration, and circulating levels of proinflammatory cytokines [97]. However, in another study, TLR4 deficiency decreased atherosclerosis but did not protect against adipocyte hypertrophy and macrophage accumulation in obese LDLR-deficient mice [98]. TLR4 also plays a central role in NASH and liver fibrosis in NAFLD. Deficiency in TLR4 expression attenuates NASH and fibrosis in mice [99]. Moreover, TLR4 deficiency in LDLR-knockout mice prevents TG accumulation and enhances FA oxidation to alter the onset of NAFLD after a high-fat/high-cholesterol diet [100]. All these results suggest that TLR4 plays an irreplaceable role in regulating obesity-related metabolic diseases.

Enhanced TLR4 signaling is increased in muscle, visceral fat tissue, and liver under obesity, which is responsible for obesity inflammation, obesity-related insulin resistance, and liver diseases [101]. Increased expression of TLR4 enhances NF-*κ*B activation and subsequently increases the release of IL-6 and TNF-*α*. In particular, released FAs by hypertrophied adipocytes activate TLR4 signaling pathways to induce inflammatory cytokines and chemokines to induce cell dysfunction in adipose tissues [102, 103]. Circulating levels of FAs are significantly higher in obese compared with lean individuals, which results in increased TLR4 signaling in obesity and inflammation [101, 103]. In obesity, the interactions between adipocytes and macrophages aggravate adipose inflammation through TLR4 [104]. M1-type macrophages highly express TLR4 and infiltrate into the target metabolic organs of obesity. By contrast, M2-type macrophages, which show a low TLR4 expression, are dominant in lean subjects. In addition, circulating levels of LPS are moderately increased in patients with obesity, T2DM, metabolic syndrome, and NAFLD [105, 106]. Increased LPS in plasma could be due to increased intestinal permeability and enhanced absorption through TLR4 by HFD [107].

In therapeutic animal studies, TAK-242 (a TLR4 inhibitor) attenuates insulin resistance in muscle cells and the adverse neural effects of diet-induced obesity [108, 109]. In an acute experiment, rats that received a TLR4 inhibitor (TAK-242 or E5564) obtain partial protection against acute and chronic fat-induced insulin resistance [110]. Berberine may reduce insulin resistance, at least in part, by modulating the gut microbiota along with inhibiting LPS-TLR4 signaling in the liver [111].

### 3.4. TLR5

TLR5 recognizes flagellin, the primary structural component of flagella, and triggers inflammatory response through MyD88. TLR5 is expressed in the intestinal mucosa against metabolic diseases, which strongly influences the composition of the microbiota throughout life [48, 112]. In loss-of-function mouse models, TLR5-deficient mice show altered gut microbiota composition, low-grade inflammation, metabolic syndrome, and predisposition to the development of colitis [113]. In mice, epithelial TLR5-mediated REG3*γ* production is critical for the counterselection of colonizing flagella [112]. TLR5-deficient mice fed a HFD also exhibit exacerbated metabolic syndromes, including hyperlipidemia, insulin resistance, and increased adiposity [114]. In addition, transferring gut microbiota from TLR5-deficient mice to WT germ-free mice confers the features of metabolic syndrome to recipients [115]. Metabolic syndrome in TLR5-deficient mice is exacerbated by HFD, suggesting that TLR5 is responsible for the balance of microbiota.

### 3.5. TLR8

There is little information about TLR8 in obesity-mediated inflammation. One study indicated that TLR8 expression in adipose tissue of nondiabetic/T2DM obese individuals is higher than that of the overweight/lean population. The elevated levels of TLR8 in obesity/T2DM are consistent with the increase of inflammatory mediators and may represent an immune marker of metabolic inflammation [116].

### 3.6. TLR9

TLR9 recognizes viral and bacterial DNA (unmethylated CpG motifs) and activates signaling through MyD88. In addition, cell-free DNA from obesity-related adipocyte degeneration can activate TLR9 [117]. A clinical association study indicated that bacterial DNA translocation to the blood is associated with metabolic dysfunction and the onset of diabetes [118]. In humans, plasma ssDNA levels are significantly high in patients with computed tomography-determined visceral obesity and are associated with homeostasis model assessment of insulin resistance [117]. The mitochondrial DNA released from hepatocytes serves as a TLR9 ligand and promotes steatohepatitis. A clinical study indicated that the mtDNA copy number is 4.3- and 3.2-fold higher in nonalcoholic fatty liver and NASH patients than in healthy controls, respectively [119].

In a loss-of-function animal study, mitochondrial DNA released by adipocytes under obesity-induced excessive stress stimulates TLR9 in the immune cells and induces inflammation in adipose tissue [120]. HFD-fed TLR9-deficient mice demonstrated reduced macrophage accumulation and inflammation in adipose tissue and better insulin sensitivity compared with WT mice, whereas bone marrow reconstitution with WT bone marrow restored the attenuation of insulin resistance observed in HFD-fed TLR9-deficient mice [117]. TLR9-deficient mice were protected against steatohepatitis due to choline-amino acid depletion and a HFD [121]. In mice, NASH development in response to a HFD required TLR9 on lysozyme-expressing cells [122].

Administration of a TLR9 inhibitory oligonucleotide to fat-fed WT mice reduced the accumulation of macrophages in adipose tissue and improved insulin resistance [117]. In mice, a TLR7/9 antagonist (IRS954) was effective for improving hepatic steatosis and NAFLD [122]. TLR9 is a target of treatments for obesity and NAFLD.

### 3.7. TLR10

A recent study indicated that TLR10 impacts adipose tissue morphology in obesity. In humans, obese individuals with polymorphisms in the TLR10 gene displayed reduced macrophage infiltration in the adipose tissue accompanied by low leptin levels and high adiponectin levels in the plasma [123]. Transgenic human TLR10 mice displayed reduced adipocyte size, adipose tissue weight, and lower plasma insulin levels compared with WT mice fed with a HFD [123]. More studies need to assess the value of TLR10 as a therapeutic target in obesity and metabolic syndrome.

## 4. NLRs and Metabolic Diseases

NLR-mediated inflammation caused by gut microbiota-derived PAMPs and metabolic stress-derived DAMPs during obesity induces metabolic disturbances and metabolic diseases (Figure 2). The NLR family contains 23 members [124]. A typical NLR is composed of three distinct common domains: the C-terminal LRR domain involved in the recognition of PAMPs and DAMPs; a centrally located NACHT that enables activation of a signaling complex via ATP-dependent oligomerization; and an N-terminal caspase recruitment domain (CARD) and PYD, which mediate downstream signaling [125].

### 4.1. NOD1/2

NOD1 and NOD2 are members of the NLR family. They are cytosolic PRRs that play a role in detecting intracellular microorganisms [126]. These receptors detect specific bacterial PGN motifs and initiate an inflammatory signaling cascade involved in host defense, which is a potential factor linking innate immunity and metabolic function [124]. NOD1 and NOD2 recognize their ligands through the LRR domain and NACHT domain oligomer by initiating downstream signaling to activate NF-*κ*B and phosphorylated MAPK, which mediate the expression of proinflammatory cytokines [124].

In a loss-of-function animal study, NOD1/2-double-deficient mice are protected from HFD-induced insulin intolerance, lipid accumulation, and inflammation in adipose tissue and liver [127]. In addition, PGN can cause acute systemic insulin resistance by activating NOD1, and NOD1-deficient mice have reduced bacterial translocation to metabolic tissues and improved insulin tolerance [128]. Moreover, NOD1 deficiency in ApoE-knockout mice showed reduced development of atherosclerotic lesions from the early stage and delayed progression of atherosclerosis [129]. However, one study showed that NOD2-deficient mice fed with a HFD have increased adipose tissue and liver inflammation and exacerbated insulin resistance [130]. This condition may be because NOD2-deficient mice have an impaired gut barrier function.

### 4.2. NLRP3 Inflammasome

As cytoplasmic complexes, inflammasomes recognize microbial and endogenous ligands and recruit procaspase-1 directly through interacting with CARD domains or indirectly through apoptosis-associated speck-like protein containing CARD (ASC), resulting in the formation of active caspase-1 and triggering the maturation of proinflammatory cytokines, such as IL-1*β* and IL-18 [125]. The NLRP3 inflammasome is activated by PAMPs from microbial pathogens, such as cell wall proteoglycans, pore-forming toxins, and DNA/RNA, and by DAMPs, such as crystals, ATP, hyaluronan, *β*-amyloid, and monosodium urate crystals. Activation of the NLRP3 inflammasome requires two steps: the first step is an NF-*κ*B-activating stimulus for cells to express pro-IL-1*β* and optimal NLRP3 and the second step is the NLRP3 inflammasome that results in caspase-1 activation [131].

Several molecules have been identified as DAMPs involved in NLRP3 activation in HFD-induced obesity. SFAs, such as palmitate and ceramides, can stimulate NLRP3 inflammasome activation to induce inflammation and obesity-mediated T2DM [132]. As an inducer of NLRP3 activation, IAPP is also accumulated in the islet of T2DM patients [31]. In addition, endocannabinoids are found to induce NLRP3 inflammasome-dependent IL-1*β* production by pancreatic infiltrating macrophages, resulting in pancreatic *β*-cell death [133]. Moreover, cholesterol crystals and oxidized LDL particles have been shown to activate the NLRP3 inflammasome, which leads to a vascular response during atherosclerosis progression [134, 135].

Human cross-sectional studies showed an increased secretion of IL-1*β*, increased expression of NLRP3, increased number of adipose tissue macrophages, and decreased number of regulatory T cells in the visceral adipose tissue of metabolically unhealthy patients compared with metabolically healthy and lean participants [136]. T2DM subjects have significantly increased mRNA and protein expression of NLRP3, ASC, and proinflammatory cytokines in monocyte-derived macrophages compared with healthy controls [137]. Weight loss in obese patients with T2DM is associated with insulin sensitivity and reduction of NLRP3 and IL-1*β* expression in adipose tissue [138]. Moreover, NLRP3 inflammasomes show high expression in the aorta of patients with atherosclerosis [139].

Animal studies provide a direct link among the NLRP3 inflammasome, chronic inflammation, and insulin resistance. A loss-of-function animal study showed that NLRP3-, ASC-, and caspase-1-deficient mice are protected from HFD-induced obesity and show improved glucose tolerance and insulin sensitivity [138, 140, 141]. NLRP3-deficient mice have reduced expression in visceral fat of markers of M1-type macrophage activation and an increase in markers of M2-type macrophage activation [138]. Gene silencing of NLRP3 suppresses atherosclerosis and stabilizes plaques in ApoE-deficient mice [142]. Liraglutide protects NAFLD via inhibiting NLRP3 inflammasome activation in a mouse model induced by HFD [143]. In addition, caspase-1 contributes to adipose tissue formation because mice lacking caspase-1 have reduced adipocyte size, reduced fat mass, increased adipogenic gene expression, and improved insulin sensitivity and gained less weight than WT controls [144]. In particular, IL-1*β* was strongly linked to the pathogenesis of T2DM by promoting insulin resistance and causing *β*-cell functional impairment and apoptosis [131]. Blockade of IL-1*β* inhibited atherosclerotic plaque formation in ApoE-deficient mice [145]. Similarly, elevated IL-18 levels have been shown to cause vascular inflammation and enhance the instability of atherosclerotic plaques, whereas IL-18-deficiency in ApoE-deficient mice results in reduced atherosclerotic lesion size [146].

A therapeutic animal study showed that MCC950, an NLRP3 selective inhibitor, improves NAFLD pathology and fibrosis in obese diabetic mice and reduces liver fibrosis in MCD-fed mice [147]. A therapeutic human study found that glyburide, a small-molecule inhibitor used for T2DM treatment, can inhibit NLRP3-dependent IL-1*β* production [148]. In addition, the IL-1 receptor antagonist (anakinra) or IL-1*β* antagonism (gevokizumab, canakizumab, and LY2189102) can effectively control glucose levels and *β*-cell function in T2DM patients [18].

### 4.3. NLRP1 Inflammasome

NLRP1 does not require ASC to activate caspase-1, which recruits procaspase-1 by directly interacting with CARD domains to trigger the maturation of IL-1*β* and IL-18. Toxoplasma infection and anthrax lethal toxin have been reported to activate NLRP1. NLRP1 functions in the context of metabolic stress to produce IL-18, preventing obesity and metabolic syndrome. NLRP1-deficient mice spontaneously develop a large increase in adipose tissue, which exacerbated by high-fat feeding and decreased IL-18 levels results in increased adiposity and resistance to diet-induced metabolic dysfunction [149].

### 4.4. NLRP6 Inflammasome

NLRP6 requires ASC to recruit procaspase-1 to activate caspase-1 and triggers the maturation of IL-1*β* and IL-18. NLRP6 plays an important function in controlling the composition of the gut microbiota in metabolic diseases [150]. Colonic microbes trigger NLRP6-mediated secretion of IL-18, which induce the expression of antimicrobial peptides that control the colonic microbial community to regulate metabolic diseases [150]. NLRP6-deficient mice are highly resistant to infection with bacterial pathogens [151]. The above results reveal that NLRP6 is a negative regulator of inflammatory signaling.

## 5. Concluding Remarks

Given the findings mentioned above, whether PRRs are part of the pathogenesis of metabolic disorders or manifestations of other derangements that arise as disorder progresses need to be investigated. The nature of inflammatory responses to obesity is unique, and many organ systems are impacted by inflammation during obesity. In many patients, insulin resistance appears long before obesity; type 1 diabetes appears and persists in the absence of obesity, and its pathogenesis likely involves entirely different immunological disturbances [152]. In this article, we only focus on the roles of PRRs underlying obesity-caused inflammation in the pathogenesis of T2DM and other related metabolic diseases.

TLRs and NLRs have been demonstrated to sense various DAMPs derived from metabolic stress or tissue damage in obesity-associated metabolic disturbances. Obesity leads to various triggering events, such as endoplasmic reticulum stress, hypoxia, and lipotoxicity, which can initiate the activation of proinflammatory pathways within metabolic tissues, including adipose tissue, liver, pancreas, gastrointestinal tract, muscle, hypothalamus, and blood vessels [101]. These receptors play critical roles in bridging immune responses and metabolic homeostasis. Thus, the development of PRR inhibitors shows huge potential as a powerful therapeutic strategy [124, 153, 154]. In general, strategies utilized to downmodulate the activation of TLRs/NLRs involve the use of antagonists, which block the binding of ligands or ligand complexes to the receptors.

At present, a number of TLR/NLR-related inhibitors have emerged [155, 156]. OPN-305, a TLR2-specific antibody, has completed clinical trials for myelodysplastic syndrome (NCT02363491 and NCT03337451). Eritoran (E5564), an antagonist of TLR4, has been terminated under clinical trials in patients with insulin resistance and T2DM for unknown reasons (NCT02321111 and NCT02267317). A small-molecule-specific inhibitor for TLR4, TAK-242 (Resatorvid), has been terminated under clinical trials in patients with sepsis-induced cardiovascular and respiratory failure due to safety and efficiency concerns (NCT00633477). A humanized monoclonal anti-TLR4 antibody (NI-0101) shows a therapeutic effect under phase-I clinical trials for LPS reaction [157]. The oligonucleotide IMO-3100 antagonizes TLR7/TLR9, and IMO-3100 has completed trials in patients with moderate to severe plaque psoriasis (NCT01622348). IMO-8400 inhibits TLR7/TLR8/TLR9 under phase-II clinical trials in patients with moderate to severe plaque psoriasis [158]. IMO-8400 has completed trials in adult patients with dermatomyositis (NCT02612857) and in patients with Waldenström's macroglobulinemia (NCT02363439). P2X7R antagonists that block the NLRP3 inflammasome are under phase-II clinical trials [159].

Although preclinical studies reported the promising inhibitory effects of some antagonistic ligands on TLR/NLR signaling, there are some challenges associated with these agents. The innate immune system is activated by PRRs and is a critical host “first responder” for exogenous pathogens and endogenous stresses. Chronic suppression may have the potential for extensive complications as implied in chronic metabolic disease. These agents mentioned above have been used in myelodysplastic syndrome, severe acute sepsis, and autoimmune diseases; only Eritoran has been administered for chronic metabolic disease. In fact, Eritoran is no longer in clinical development due to either clinical or nonclinical toxicity or lack of efficacy.

Additional studies are required to provide new insights into the therapeutic targeting of TLRs/NLRs, and the development of new screening methods and identification of drugs with good pharmacology is important. To date, the involvement of TLRs/NLRs in the development of inflammatory diseases continues to be unveiled, which indicates that the overactivation of these receptors causes disruption of homeostasis, increasing the risk for metabolic diseases. Thus, TLR/NLR-driven responses should be tightly regulated to prevent any detrimental effect from their aberrant activation.
